# Inhibition effects and binding interactions of epigallocatechin and gallocatechin on tyrosinase and their anti-melanogenesis activity

**DOI:** 10.1080/14756366.2026.2716559

**Published:** 2026-08-26

**Authors:** Jianjun Zhang, Zheng Shi, Jiang Li, Kaiming Li, Fan Wu, Min Zhao, Rui Xie, Hongbin Luo

**Affiliations:** The First Affiliated Hospital of Zhejiang Chinese Medical University (Zhejiang Provincial Hospital of Chinese Medicine), Hangzhou, China

**Keywords:** Tyrosinase, multi-spectroscopic, inhibition mechanisms, epigallocatechin, gallocatechin

## Abstract

Tyrosinase is a key enzyme in melanogenesis and food enzymatic browning. This study systematically investigated tyrosinase inhibition effects and binding interactions of two tea catechins epigallocatechin (EGC) and gallocatechin (GC), and their practical efficacy. *In vitro* enzymatic assays showed that EGC and GC both reversibly inhibited tyrosinase in a mixed-type manner, with IC_50_ values of 0.059 ± 0.002 mg/ml (192.02 ± 7.19 µM) and 0.036 ± 0.001 mg/ml (118.77 ± 4.11 µM), respectively. Fluorescence quenching, synchronous fluorescence, CD spectra, ANS-binding assay, and molecular docking results revealed the binding between EGC or GC and tyrosinase, changed enzyme conformation and microenvironment, subsequently leading to a decrease in enzyme activity. Cellular studies demonstrated that EGC and GC obviously inhibited intracellular tyrosinase activity and melanin synthesis in B16 melanoma cells. These findings provide comprehensive mechanistic insights into the anti-tyrosinase activity of EGC and GC and support their potential application as natural inhibitors.

## HIGHLIGHTS

1. Tyrosinase inhibitory effects and mechanisms of epigallocatechin and gallocatechin were firstly investigated.
2. The binding of epigallocatechin and gallocatechin with tyrosinase caused changes in enzyme conformation and microenvironment, leading to a decrease in enzyme activity.
3. Epigallocatechin and gallocatechin showed anti-melanogenesis efficacy.

## Introduction

Tyrosinase is a ubiquitous copper-dependent oxidase, pivotal in the biosynthesis of melanin across various biological systems^1–3^. Its catalytic function involves the hydroxylation of monophenols and the oxidation of diphenols, initiating the cascade that culminates in melanin formation^4–6^. In mammalian skin, the overexpression or hyperactivation of this enzyme is directly associated with hyperpigmentation disorders, including melasma, age spots, and melanoma^7–9^. Beyond dermatology, the enzymatic browning catalysed by tyrosinase presents a significant challenge in the food industry, leading to nutrient loss and shortened shelf-life in fresh-cut fruits and vegetables^10–12^. Consequently, the identification and characterisation of effective tyrosinase inhibitors are of considerable interest for both cosmetic therapeutics and food preservation technologies^13^^,^^14^.

**Figure 1.**
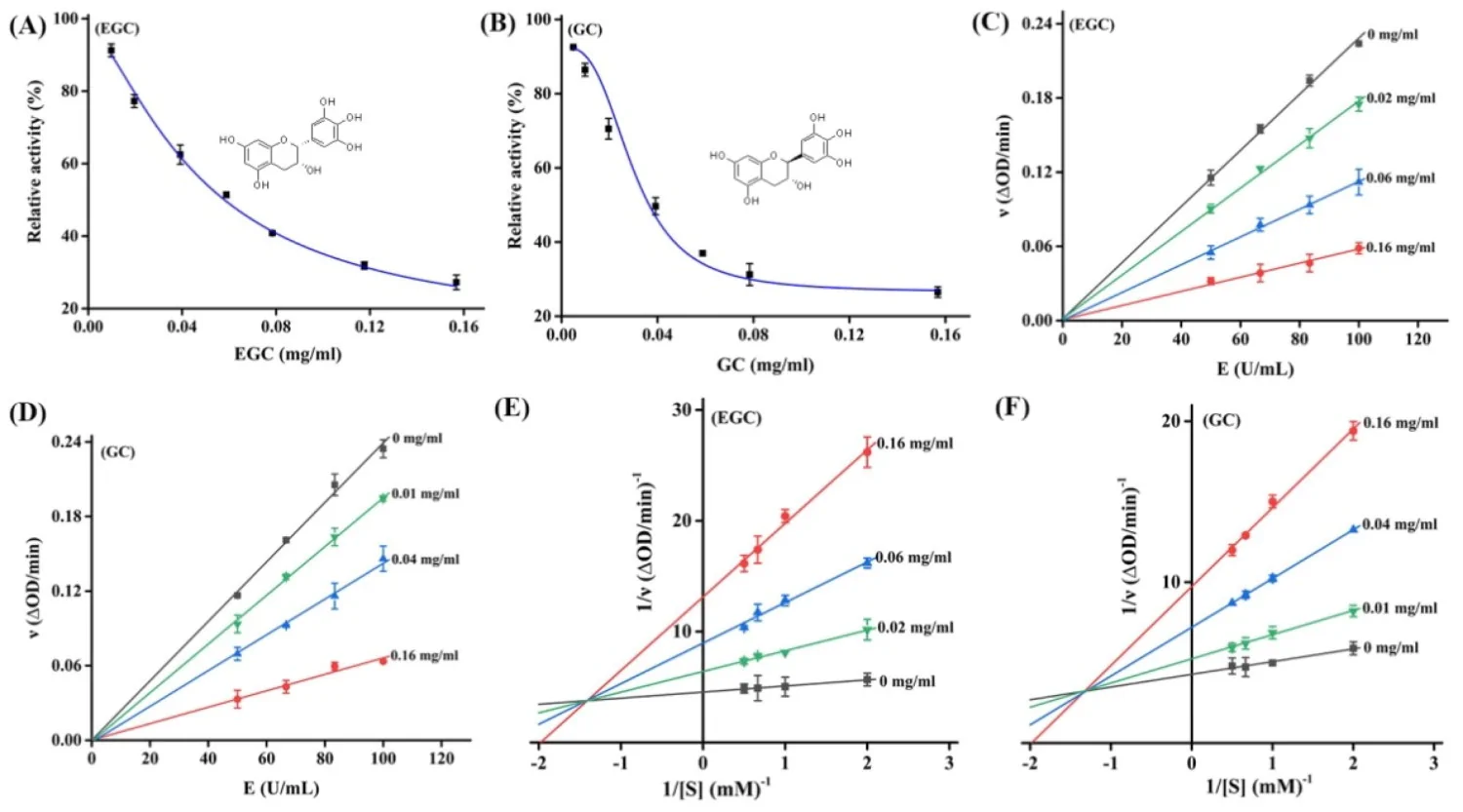
(A-B) Inhibition of EGC and GC on tyrosinase; (C-D) Reversibility assay of EGC and GC; (E-F) Inhibition type assay of EGC and GC.

There are abundant types of natural products^15–19^, and a large number of active ingredients have been obtained, such as polysaccharides^20–24^, peptides^25^, active small molecules^26^, etc. These active ingredients exhibit rich biological activities, such as anti-tumor^27–29^, antioxidant^30^, and anti-alzheimer^31^^,^^32^. Therefore, natural active small molecules have become an important source of clinical drugs^33–37^. In addition, many active molecules based on natural products have also been developed^38–42^, including tyrosinase inhibitors^43–45^. Among natural products, catechins, a predominant subclass of tea polyphenols, are renowned for their broad spectrum of bioactivities, encompassing antioxidant, anti-inflammatory, and anti-obesity properties^46^. Catechins also demonstrate potential tyrosinase inhibitory activity. Among these, epigallocatechin gallate (EGCG) and gallocatechin gallate (GCG) have been investigated in detail for their potent tyrosinase inhibitory effects^47^^,^^48^. In contrast, their corresponding precursor molecules, epigallocatechin (EGC, Figure 1A) and gallocatechin (GC, Figure 1B) have received comparatively scant attention regarding their direct inhibitory effects and underlying mechanisms on tyrosinase. Preliminary evidence suggests that crude extracts containing EGC and GC possess anti-tyrosinase and anti-melanogenesis activities^49^. As a pair of diastereomers (EGC and GC), their inhibition effects and interaction mechanisms against tyrosinase, and the effects of structural differences on their tyrosinase inhibition is lacking.

Therefore, this study aims to elucidate the inhibitory effects and mechanisms of EGC and GC against tyrosinase through integrated multi-technique approaches, including kinetics, multi-spectra, and molecular docking^50^. Furthermore, the practical efficacy of EGC and GC is validated through cellular anti-melanogenesis assays in B16 cells.

## Materials and methods

### Materials

EGC (purity ≥ 98%) and GC (purity ≥ 98%) were obtained from Yuanye Biotechnology Co. (Shanghai, China). Tyrosinase from mushroom was purchased from Sigma-Aldrich. Levodopa (L-Dopa) were obtained from Bidepharm. All other reagents were analytical grade and acquired from commercial sources.

### Inhibitory on tyrosinase

The inhibitory effects of EGC and GC on tyrosinase were determined using L-DOPA as substrate^51^. Briefly, tyrosinase (final concentration 0.67 U/ml) was pre-incubated with varying concentrations of EGC or GC at room temperature for 5 min. The reaction was initiated by adding L-Dopa (final concentration 0.5 mM). The mixture was monitored by measuring absorbance change at 475 nm. The percentage inhibition was calculated based on the blank.

### Kinetic studies

To investigate the inhibitory reversibility and type, enzyme kinetic studies were conducted^52–54^. For reversibility assessment, a series of reaction mixtures containing varying concentrations of EGC (0, 0.02, 0.06, 0.16 mg/ml) or GC (0, 0.02, 0.06, 0.16 mg/ml), and fixed L-Dopa concentration (0.5 mM) were prepared. The enzymatic reaction rate (ΔA/min) was measured at different tyrosinase concentrations (0.50, 0.67, 0.84, 1.00 U/ml). The linear relationship between reaction velocity and enzyme concentration was analysed graphically.

For inhibitory type, the reaction rates were monitored with increasing concentrations of L-Dopa (0.50, 1, 1.25, 2 mM) in different condition of EGC (0, 0.02, 0.06, 0.16 mg/ml) or GC (0, 0.02, 0.06, 0.16 mg/ml), and tyrosinase (0.67 µM). Lineweaver-Burk plots were used to analyse the obtained data^9^^,^^55^. The inhibition type was identified based on the pattern of line intersections. Subsequently, the dissociation constants for the inhibitor binding to the free enzyme (*K*_i_) and the enzyme-substrate complex (*K*_is_) were calculated by secondary plots of the slope or intercept versus inhibitor concentration^56^.

### Fluorescence quenching

The interaction between EGC/GC and tyrosinase was investigated using fluorescence spectra at 295, 295, and 301 K, respectively, according to previous methods^57–59^. A fixed concentration of tyrosinase (0.17 mg/ml) was titrated with successive aliquots of EGC or GC solutions (0, 2.5, 5, 7.5, 10, 12.5, 15, 17.5 μM). Fluorescence spectra were recorded under excitation wavelength of 280 nm.

### Synchronous fluorescence spectra

Synchronous fluorescence (SF) spectra were performed to probe microenvironment changes around specific aromatic amino acid residues in tyrosinase^60–62^. A fixed concentration of tyrosinase (0.17 mg/ml) was titrated with increasing concentrations of EGC or GC. SF spectra were acquired at wavelength intervals (Δλ) of 15 nm (characteristic of tyrosine residues) and 60 nm (characteristic of tryptophan residues).

### Circular dichroism

Circular dichroism (CD) spectra were employed to evaluate the potential influence of EGC and GC binding on the secondary structure of tyrosinase^63–65^. 100 μL of mixture with different molar ratios of tyrosinase to EGC or GC were prepared by adding EGC or GC into tyrosinase solution. Then, the CD spectra were monitored.

### 8-Anilino-1-naphthalenesulfonic acid binding assay

8-Anilino-1-naphthalenesulfonic acid (ANS) probe was assessed to assay surface hydrophobicity changes of tyrosinase upon interaction with EGC or GC^66–68^. Briefly, tyrosinase (0.1 mg/mL) was incubated with ANS (80 µM) in the dark for 30 min to allow the probe to bind to hydrophobic surface. Subsequently, the pre-formed complex was titrated with increasing concentrations of EGC or GC (0, 2.5, 5, 7.5, 10, 12.5, 15, 17.5 μM). Fluorescence spectra were recorded immediately after each addition at excitation wavelength of 350 nm.

### Molecular docking

Molecular docking studies were performed to predict the binding interactions of EGC or GC with tyrosinase using AutoDock 4.2^69–73^. Tyrosinase structure (PDB: 2Y9X) was retrieved from the RCSB Protein Data Bank. Tyrosinase was prepared using standard protocols, which included the removal of water molecules and ligands, followed by the addition of polar hydrogen atoms and charges. EGC and GC structures were constructed in Chem3D software and energy-minimized using SYBYL software. Docking simulations were carried out in default mode.

### Cellular assay

#### Intracellular tyrosinase activity

The inhibitory effects of EGC and GC on intracellular tyrosinase activity was determined in B16 cells^74^. After cells (10^5^ cells/well) were seeded in 6-well plates for 24 h, cells were treated with EGC or GC (0.01, 0.02, 0.04 mg/ml) for 48 h. Following treatment, cells were washed, lysed with 1% Triton X-100 solution under condition of −80 °C. The lysates were centrifuged, and the protein concentration of the supernatant was quantified. An aliquot of the supernatant containing equal amounts of protein was incubated with L-Dopa (2 mg/mL) at 37 °C. Dopachrome formation rate was monitored by measuring the absorbance at 475 nm. Intracellular tyrosinase activity was expressed relative to that of untreated control cells.

#### Melanin inhibition

The effects of EGC and GC on melanogenesis was evaluated by quantifying cellular melanin content^75^^,^^76^. B16 cells were seeded and treated as described in above section. The harvested cells were boiled in NaOH (1 M) for 1 h to dissolve melanin. The absorbance was measured at 405 nm. Melanin inhibition was expressed as a percentage relative to untreated control cells.

### Statistical analysis

Data were presented as mean ± SD. One-way ANOVA was performed to assay the difference between groups. *P* < 0.05 was considered significant^77^^,^^78^.

## Results and discussion

### Inhibitory potency of EGC and GC on tyrosinase

The inhibitory effects of EGC and GC on mushroom tyrosinase activity were quantitatively evaluated. As illustrated in Figure 1A,B, both EGC and GC displayed a concentration-dependent suppression on the tyrosinase activity with IC_50_ values of 0.059 ± 0.002 mg/ml (192.02 ± 7.19 µM) and 0.036 ± 0.001 mg/ml (118.77 ± 4.11 µM), respectively. The inhibitory activities of EGC and GC were considerably weaker than that of the positive control kojic acid (IC_50_ = 16.89 ± 2.04 µM), indicating that EGC and GC were moderate potency tyrosinase inhibitors. EGC and GC are isomers of each other with cis/trans conformation. The SAR analysis revealed that GC with trans-configuration at the C-2 position showed 1.6-fold stronger tyrosinase inhibitory potency than EGC with cis-configuration at the C-2 position. This difference was likely attributable to the more favourable spatial orientation of the B-ring hydroxyl groups in the trans-configuration, which might facilitate stronger interactions with tyrosinase, whereas the cis-configuration of EGC might impose greater steric constraints that hindered optimal binding.

### Reversibility

To determine the inhibition characteristic of EGC and GC, the relationship between enzymatic reaction velocity and tyrosinase concentration was analysed in the presence of EGC or GC, respectively. As depicted in Figure 1C,D, plots of the initial reaction rate *versus* enzyme concentration yielded a family of straight lines that all converged at the origin. Furthermore, the slopes of these lines decreased systematically with increasing inhibitor concentration. This linear pattern intersecting at the origin was the typical characteristic of reversible inhibition. The results confirmed that both EGC and GC reversibly interacted with tyrosinase, forming a dynamic complex, to reduce its catalytic efficiency.

### Inhibition type

The inhibition types of EGC and GC were elucidated using kinetics analysis. Lineweaver-Burk plots were constructed from reaction rates measured at varying substrate concentrations in presence of EGC or GC, respectively. As shown in Figure 1E,F, the resulting linear plots intersected in the second quadrant for both EGC and GC. With increasing concentrations of EGC or GC, the apparent maximum velocity (V_max_) decreased, while the apparent Michaelis constant (K_m_) increased. This pattern of concurrent changes in both kinetic parameters suggested mixed-type inhibition type of EGC and GC on tyrosinase. As mixed-type inhibitors, EGC and GC could bind to both free tyrosinase and tyrosinase-substrate complex, thus, inhibiting the enzyme activity. The Slopes of Lineweaver-Burk plots increased with increasing inhibitor concentrations (EGC: 0.55, 1.87, 3.66, and 6.63; GC: 0.79, 1.50, 3.00, and 4.90). Secondary plots of slope *versus* inhibitor concentration were constructed to derive the dissociation constants K_i_ (for free enzyme), yielding values of 0.03 mg/ml for EGC and 0.06 mg/ml for GC. Similarly, secondary plots of intercept *versus* inhibitor concentration provided the dissociation constants (K_is_) for binding to the enzyme-substrate complex, which were 0.10 mg/mL for EGC and 0.16 mg/mL for GC. The K_i_ values of both EGC and GC were obviously lower than their corresponding K_is_ values, indicating that EGC and GC preferred to bind onto free enzyme than enzyme-substrate complex.

### Fluorescence quenching

**Figure 2.**
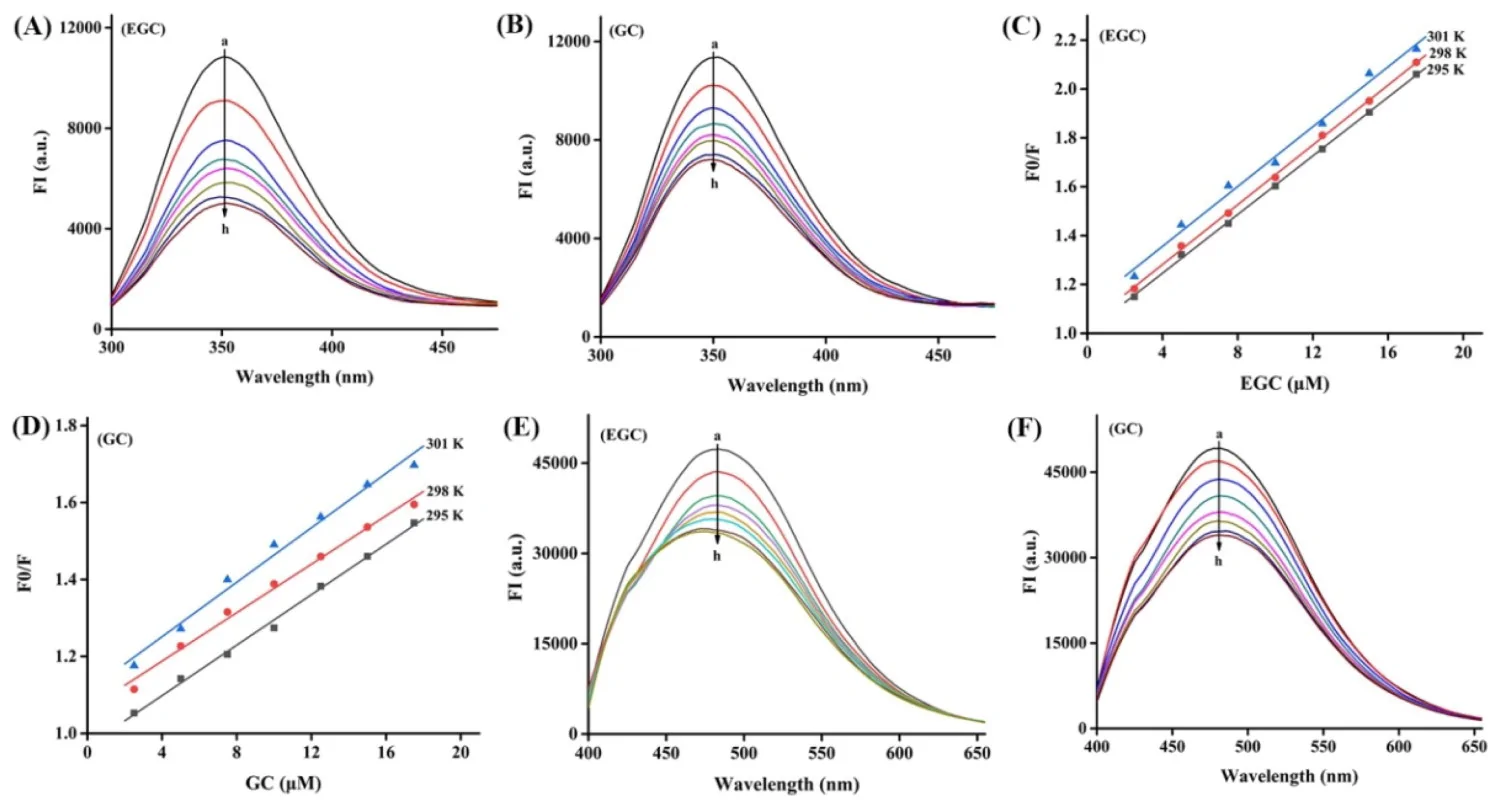
(A-B) Fluorescence quenching of tyrosinase by EGC and GC; (C-D) Stern-Volmer plots of EGC and GC. (E-F) ANS-binding assay of EGC and GC.

The molecular interactions between EGC and GC and tyrosinase were further investigated through fluorescence quenching. As illustrated in Figure 2A,B, tyrosinase exhibits a strong intrinsic fluorescence emission peak when excited at 280 nm, primarily attributed to its tryptophan and tyrosine residues. However, the progressive addition of either EGC or GC to tyrosinase resulted in a concentration-dependent decrease in fluorescence intensity at 295 K (Figure 2A,B). The phenomena revealed the binding of EGC or GC with tyrosinase, thereby, quenching the fluorescence.

To elucidate the underlying quenching mechanism, the quenching experiments were conducted at 295, 298, and 301 K, respectively, which were analysed using the Stern-Volmer equation. The Stern-Volmer plots exhibited good linearity for both EGC and GC (Figure 2C,D), suggesting the single quenching process. The calculated bimolecular quenching rate constants (K_q_) for both EGC and GC were on the order of 10^12^ L/(mol·s) (Table 1), which were obvious higher than the maximum diffusion-controlled collision constant (2.0 × 10^10^ L/(mol·s)). Moreover, the Stern-Volmer constant (K_SV_) decreased with increasing temperature (Table 1). Above results strongly indicated the static quenching mechanism of both EGC and GC against tyrosinase, which also implied the formation of a stable non-fluorescent ground-state complex between EGC and GC and tyrosinase.

**Table 1.** Quenching parameters of EGC and GC on tyrosinase.

	T (K)	K_q_ (×10^12^ L/(mol·S)	K_SV_ (×10^4^ L/mol)	K_a_ (×10^4^ L/mol)	n
EGC	295	6.12	6.12	5.56	0.99
	298	6.12	6.12	2.52	0.92
	301	6.00	6.00	0.86	0.81
GC	295	3.28	3.28	22.18	1.18
	298	3.15	3.15	0.58	0.83
	301	3.04	3.04	0.21	0.73

### Binding affinity and stoichiometry

The equilibrium binding parameters between EGC and GC with tyrosinase were further derived from the fluorescence quenching data by modified Stern-Volmer equation. The binding constant (K_a_), which reflects the affinity of inhibitor on enzyme, and the binding sites number (n) were calculated. As summarised in Table 1, K_a_ values for the complexes were on the order of 10^4^, which indicated moderate binding affinity for both EGC and GC to tyrosinase under the experimental conditions. The n values for both EGC and GC were approximately 1.0, suggesting that each tyrosinase molecule possessed a single primary binding site for both EGC and GC.

### Thermodynamic parameters

To identify the dominant intermolecular forces stabilising the inhibitor-tyrosinase complex, key thermodynamic parameters (Table 2) were obtained using the van’t Hoff equation. The calculated ΔG values for both EGC and GC were negative, confirming that their binding processes were spontaneous. The ΔH and ΔS values were both negative for the formation of each complex, which were classically indicative of binding interactions driven primarily by hydrogen bonding and van der Waals forces. That was to say that polar interactions and close-range van der Waals contacts played crucial roles in complex stabilisation and inhibitor specificity.

**Table 2.** Thermodynamic parameters of EGC and GC on tyrosinase.

	T (K)	△H (KJ/mol)	△G (KJ/mol)	△S (J/(mol·K)
EGC	295	−137.66	−27.62	−373.06
	298		−25.38	
	301		−23.14	
GC	295	−130.08	−30.20	−338.60
	298		−29.18	
	301		−28.17	

### ANS-binding assay

The effects of EGC and GC binding on the surface hydrophobicity of tyrosinase were investigated using the hydrophobic fluorescent probe ANS. As shown in Figure 2E,F, the fluorescence intensity of tyrosinase-ANS decreased progressively with increasing EGC (Figure 2E) and GC (Figure 2F). These reduction in ANS fluorescence indicated a decrease in the accessibility of hydrophobic patches on the enzyme surface. This phenomenon might be due to the direct occupancy of surface-exposed hydrophobic cavities by EGC or GC, and ligand-induced conformation changes.

### SF spectra

**Figure 3.**
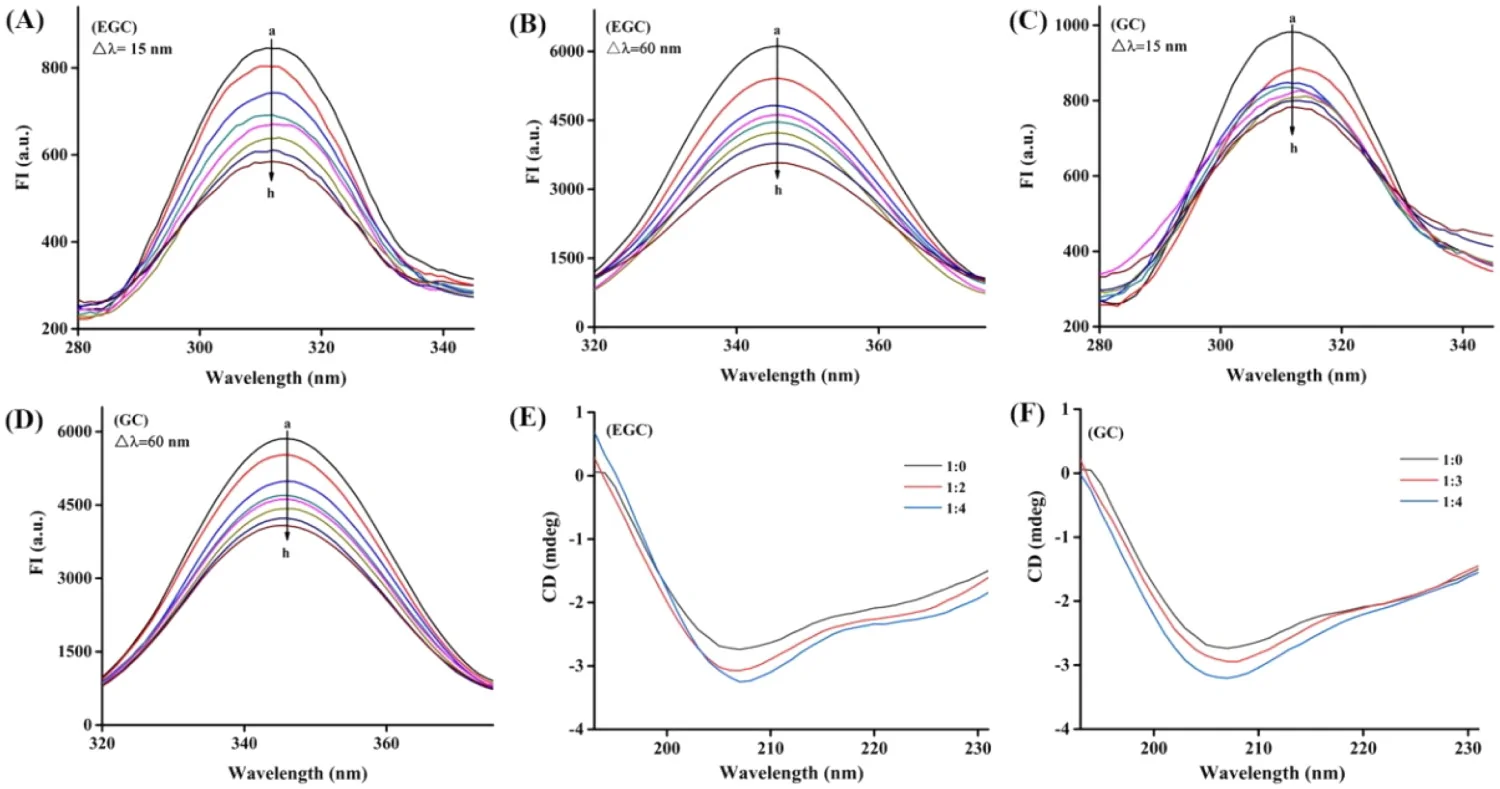
(A-B) SF spectra of tyrosinase by EGC at Δλ of 15 and 60 nm; (C-D). SF spectra of tyrosinase by GC at Δλ of 15 and 60 nm. (E-F) CD spectra of tyrosinase upon EGC and GC.

SF spectra were employed to probe the microenvironments of specific aromatic amino acid residues within tyrosinase upon binding with EGC or GC. SF spectra of tyrosine (Tyr) and tryptophan (Trp) were recorded at Δλ of 15 and 60 nm, respectively. The addition of either EGC (Figure 3A,B) or GC (Figure 3C,D) led to a progressive decrease in SF intensity of both Tyr and Trp residues. However, maximum emission peaks did not display an obvious shift with the increase of EGC or GC concentration. These results implied that while EGC and GC interacted with these aromatic residues, they showed weak effects on the microenvironment around Tyr and Trp residues. This phenomenon partly aligned with our fluorescence quenching results.

### CD spectra

CD spectra were utilised to examine potential conformation changes in the secondary structure of tyrosinase induced by EGC or GC binding. CD spectra of free tyrosinase (Figure 3E,F) exhibited characteristic negative bands at approximately 208 nm and 222 nm, indicative of a predominantly α-helical structure. Upon the addition of EGC or GC, obvious alterations in spectral profile were observed (Figure 3E,F), revealing the effects of EGC or GC binding. The secondary structure contents were obtained from CD spectra data (Table 3), which revealed the specific changes. For EGC, the addition led to increases in α-helix and β-turn contents, with a concomitant decrease in β-sheet and random coil contents. In contrast, the binding of GC caused increases in α-helix and random coil contents, and reduction in β-turn and β-sheet contents. Usually, enzyme inactivation is associated with a decrease in α-helix, while our result showed an increase in α-helix, which might be due to local stiffening of specific peptide segments near the active site during ligand binding. Similar phenomena have also been found in Ar-turmerone binding tyrosinase^79^. These changes suggested the interactions of both compounds with tyrosinase perturbed its native folding, leading to the loss of enzymatic activity.

**Table 3.** The secondary structure contents of tyrosinase upon EGC or GC (%).

	Molar ratio	α-Helix	β-Sheet	β-Turn	Random Coil
EGC	1:0	12.70	47.20	20.90	31.00
	1:2	12.80	46.60	21.00	31.20
	1:4	12.90	46.30	21.20	31.40
GC	1:0	13.60	47.20	21.50	32.70
	1:3	13.70	47.10	21.40	32.80
	1:4	13.80	46.90	21.30	32.90

### Molecular docking

**Figure 4.**
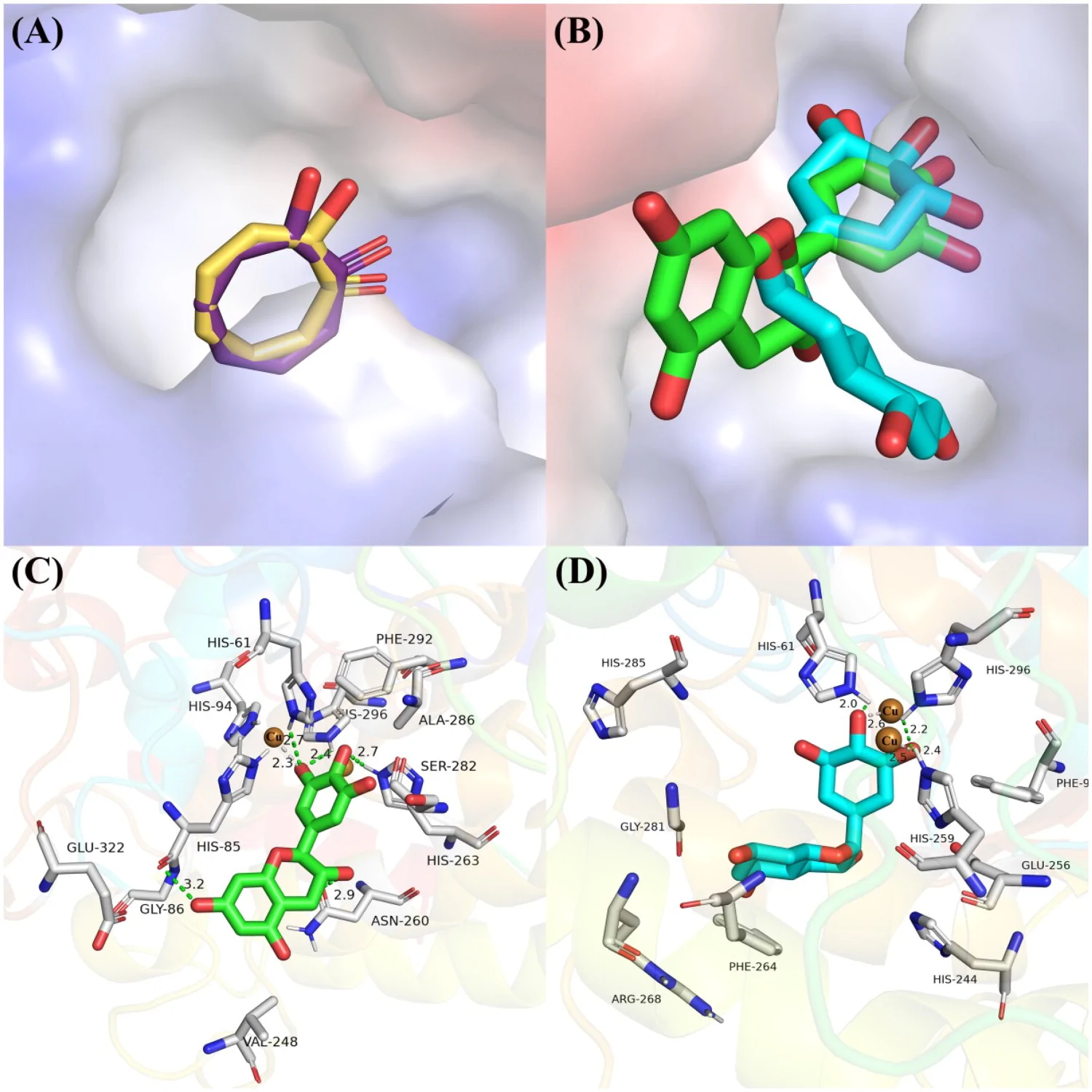
(A) The binding conformation of tropolone; (B) Docking conformation of EGC and GC in tyrosinase active site; (C-D) Docking interactions of EGC and GC onto tyrosinase.

Molecular docking was used to simulate the binding interactions between EGC and GC with tyrosinase. For validating docking protocol, the protein ligand, tropolone was also docked. As shown in Figure 4A, tropolone conformation was well superposed to that in the crystal structure (RMSD < 1.0 Å). EGC and GC were also well docked into tyrosinase active site with conformation differences on chromone rings of EGC and GC (Figure 4B). EGC formed hydrogen bonds with HIS-61 (2.0 Å), HIS-259(2.4 Å), and HIS-296 (2.2 Å), hydrophobic interactions with PHE-90, HIS-244, GLU-256, PHE-264, ARG-268, GLY-281, and HIS-285, and chelation interactions with Cu^2+^ (2.5 and 2.6 Å) (Figure 4C). GC formed hydrogen bonds with HIS-61 (2.7 Å), HIS-85(3.2 Å), ASN-260(2.9 Å), HIS-263 (2.7 Å), and HIS-296 (2.4 Å), and hydrophobic interactions with GLY-86, HIS-94, VAL-248, SER-282, ALA-286, PHE-292, and GLU-322, and chelation interactions with Cu^2+^ (2.3 and 2.6 Å) (Figure 4D). As isomers, EGC and GC differed at cis/trans configuration at C-2 position. Activity results showed that GC showed 1.6-fold stronger tyrosinase inhibitory potency than EGC. GC formed five hydrogen bonds (involving His-61, His-85, Asn-260, His-263, and His-296) with tyrosinase, whereas EGC formed only three (His-61, His-259, and His-296). Moreover, the Cu^2+^ chelation distances for GC (2.3 and 2.6 Å) were slightly shorter than those for EGC (2.5 and 2.6 Å), indicating stronger metal coordination. These findings were consistent with the experimental IC_50_ data, collectively supporting that the trans-configuration at C-2 enables more extensive hydrogen-bonding networks and more favourable copper chelation, thereby conferring higher inhibitory potency to GC. From a pharmacophore perspective, the C-2 trans-configuration and the spatial orientation of B-ring hydroxyl groups emerged as key structural features favouring tyrosinase inhibition.

### Molecular docking dynamics

**Figure 5.**
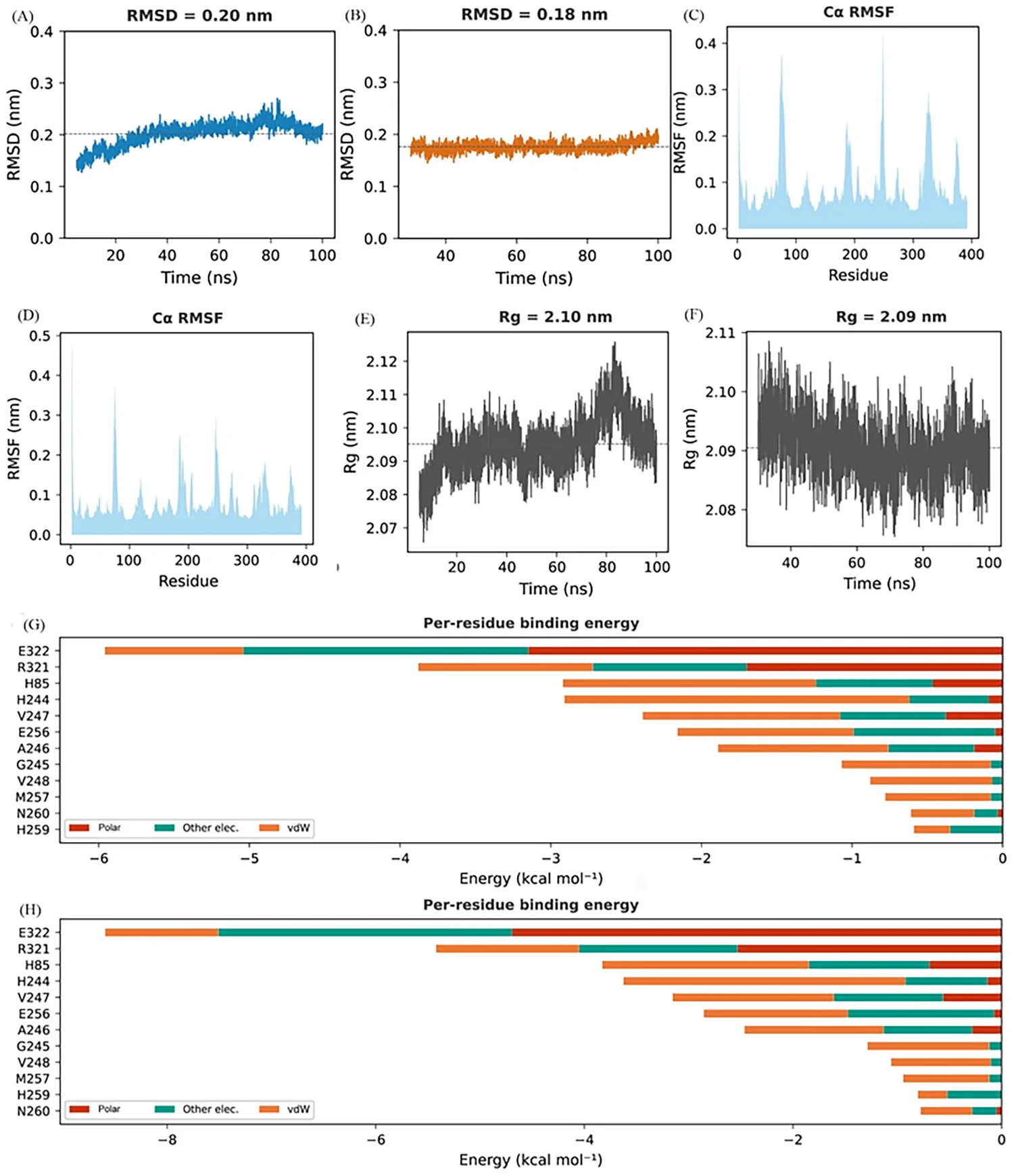
Molecular docking dynamics of GC and EGC on tyrosinase. (A-B) RMSD of 378 GC and EGC; (C-D) RMSF of GC and EGC; (E-F) Rg of GC and EGC; (G-H) 379 Pre-residue binding energy of GC and EGC.

The binding behaviour of GC and EGC at tyrosinase active site was examined over 100-ns all-atom MD trajectories. The ligand RMSD averaged 0.201 ± 0.023 nm for GC and 0.176 ± 0.009 nm for EGC (Figure 5A,B). GC showed a gradual early increase towards a plateau near 0.20 nm and a broader, partly bimodal RMSD distribution, whereas EGC remained more narrowly distributed around 0.18 nm. The Cα RMSF profiles were also similar overall (Figure 5C,D), although GC displayed a prominent internal peak near residue 248, whereas the largest EGC fluctuation occurred at the N terminus. The radius of gyration was stable in both systems (GC: 2.095 ± 0.008 nm; EGC: 2.090 ± 0.005 nm; Figure 5E,F), indicating no appreciable global compaction or expansion.

The binding energy between GC and key amino acids was further evaluated. GC occupied the substrate-binding cavity adjacent to the dinuclear copper site and was stabilised by a combination of polar, other electrostatic, and van der Waals interactions (Figure 5G). GLU322 was the largest per-residue contributor (approximately −5.96 kcal/mol). ARG321 was the second-largest contributor (approximately −3.88 kcal/mol). HIS85 contributed approximately −2.91 kcal/mol. VAL247 contributed approximately −2.36 kcal/mol, and GLU256, ALA246, GLY245, VAL248, MET257, ASN260, and HIS259 made additional smaller contributions. For EGC, the ranked residues in the energy-decomposition panel were the same as for GC, with GLU322, ARG321, HIS85, HIS244, and VAL247 forming the leading group. GLU322 made the most favourable EGC contribution (approximately −8.59 kcal/mol). ARG321 also contributed more favourably in the EGC system (approximately −5.35 kcal/mol).

The 100-ns trajectories showed that both GC and EGC remained stably associated with the copper-adjacent pocket. GLU322 was the dominant local anchor in both complexes, while C3 stereochemistry changed the accessory contact network.

### ADMET assay

The pharmacological properties of EGC and GC were analysed using SwissADME software. As shown in Table 4, EGC and GC both showed good Lipinski’s Rule of Five, including molecular weight, rotatable bonds, H-bond acceptors, molar refractivity, polar surface area, and bioavailability score. However, since EGC and GC were isomers of each other with cis/trans configuration at the C-2 position, their predicted properties were the same.

**Table 4.** ADMET properties of EGC and GC.

Parameter	GC	EGC
Molecular weight (g/mol)	306.27	306.27
Rotatable bonds	1	1
H-bond acceptors	7	7
H-bond donors	6	6
Molar refractivity	76.36	76.36
Polar surface area (Å²)	130.61	130.61
Bioavailability score	0.55	0.55

### C*ellular activity*

**Figure 6.**
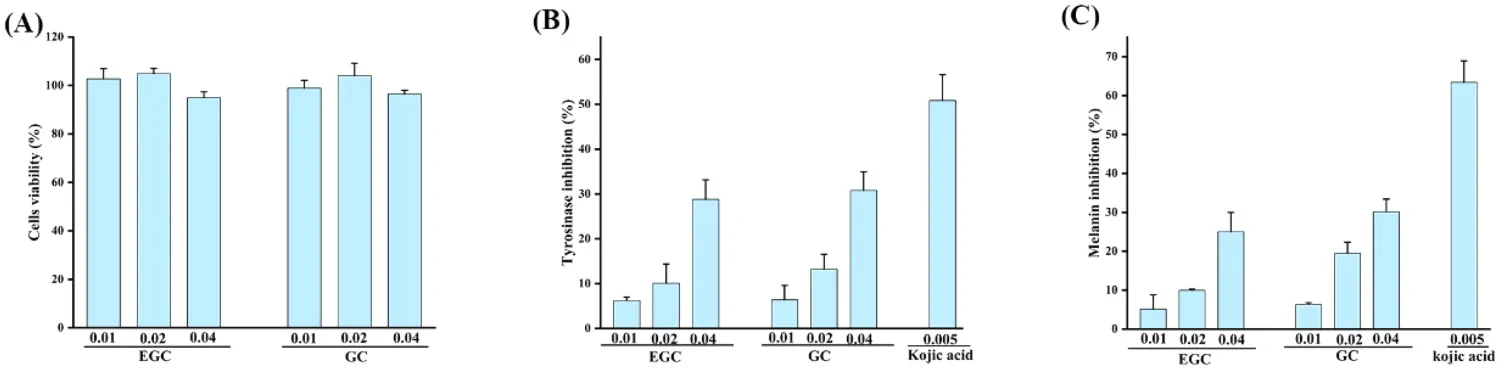
(A) Cells viability of EGC and GC; (B) Intracellular tyrosinase inhibition of EGC and GC; (C) Melanin inhibition of EGC and GC.

Firstly, the B16 cells viabilities under EGC and GC were tested and results showed that EGC and GC showed no toxicity under 0.01–0.04 mg/ml (Figure 6A). Subsequently, the effects of EGC and GC on intracellular tyrosinase activity and melanogenesis were examined in B16 cells. As presented in Figure 6B,C, both EGC and GC (0.01–0.04 mg/ml) demonstrated a concentration-dependent inhibition on cellular tyrosinase activity, as well as corresponding reduction in melanin content. However, the EGC and GC showed weaker inhibitory effects on cellular tyrosinase activity and melanin content compared to control kojic acid (0.005 mg/ml) (Figure 6B,C). These intracellular results corroborated *in vitro* enzyme inhibition activity of EGC and GC. Especially, these results confirmed that EGC and GC could exert their anti-tyrosinase function and subsequently alleviating melanin production within a physiological environment.

## Conclusion

This study provided a comprehensive investigation into the inhibition of mushroom tyrosinase by two catechins, EGC and GC. The results demonstrated that both EGC and GC acted as reversible and mixed-type inhibitors, with moderate *in vitro* potency (IC_50_ = 0.059 ± 0.002 and 0.036 ± 0.001 mg/ml). Through an integrated approach combining enzyme kinetics, multi-spectroscopic, and computational modelling, we elucidated the underlying interaction mechanisms. EGC and GC formed stable ground-state complexes with tyrosinase, primarily driven by hydrogen bonding and van der Waals forces. Binding induced conformation changes, reducing the enzyme’s structural compactness and surface hydrophobicity. Molecular docking revealed that both inhibitors occupied the catalytic pocket, interacting with key copper-coordinating and substrate-recognition residues. Especially, both catechins effectively suppressed intracellular tyrosinase activity and melanin synthesis in B16 melanoma cells. In summary, this work not only clarified the molecular interaction mechanisms of EGC and GC with tyrosinase but also validated their dual-function potential as natural agents for managing hyperpigmentation.

## Data Availability

The datasets presented in current study are available from the corresponding author upon reasonable request.
